# Review of Antimicrobial Resistance in the Environment and Its Relevance to Environmental Regulators

**DOI:** 10.3389/fmicb.2016.01728

**Published:** 2016-11-01

**Authors:** Andrew C. Singer, Helen Shaw, Vicki Rhodes, Alwyn Hart

**Affiliations:** ^1^NERC Centre for Ecology & HydrologyWallingford, UK; ^2^Department for Environment, Food and Rural AffairsLondon, UK; ^3^Environment AgencyBristol, UK

**Keywords:** antimicrobial resistance, AMR, antibiotics, metals, biocide, plasmid, genes

## Abstract

The environment is increasingly being recognized for the role it might play in the global spread of clinically relevant antibiotic resistance. Environmental regulators monitor and control many of the pathways responsible for the release of resistance-driving chemicals into the environment (e.g., antimicrobials, metals, and biocides). Hence, environmental regulators should be contributing significantly to the development of global and national antimicrobial resistance (AMR) action plans. It is argued that the lack of environment-facing mitigation actions included in existing AMR action plans is likely a function of our poor fundamental understanding of many of the key issues. Here, we aim to present the problem with AMR in the environment through the lens of an environmental regulator, using the Environment Agency (England’s regulator) as an example from which parallels can be drawn globally. The issues that are pertinent to environmental regulators are drawn out to answer: What are the drivers and pathways of AMR? How do these relate to the normal work, powers and duties of environmental regulators? What are the knowledge gaps that hinder the delivery of environmental protection from AMR? We offer several thought experiments for how different mitigation strategies might proceed. We conclude that: (1) AMR Action Plans do not tackle all the potentially relevant pathways and drivers of AMR in the environment; and (2) AMR Action Plans are deficient partly because the science to inform policy is lacking and this needs to be addressed.

## Introduction

Many of the hurdles to combating antibiotic-resistant infections in the clinic are well understood and, as such, have been used to inform existing antimicrobial resistance (AMR) Action Plans (European Commission, 2011; Department of Health/Defra, 2013; World Health Organisation, 2015). It is argued, that our inability to satisfactorily answer fundamental questions about AMR in the environment is responsible for the lack of any significant environmental focus in existing AMR Action Plans and the O’Neill AMR Reviews (O’Neill and The Review on Antimicrobial Resistance, 2016). It is further argued that without inclusion or consideration of all the drivers and pathways of AMR into the environment, AMR Action Plans are incomplete and at risk of not achieving the desired goals of ensuring and improving the efficacy of existing and future antibiotics.

In this review, we aim to present the AMR challenge through the lens of an environmental regulator, using the Environment Agency (England’s regulator) as an example from which parallels can be drawn globally. We argue that there is an evidence gap that hinders the ability of policymakers and environmental regulators from delivering environmental protection from AMR. The following five questions exemplify the approach taken by an environmental regulator when tackling the AMR challenge. The inability to answer these questions, in this point in time, highlights some of the evidence gaps that need to be prioritized to facilitate a holistic, evidence-based AMR Action Plan:

(1) What are the benefits of controlling AMR in the environment over and above mitigating the potential transmission of resistance to humans?
(2) What is the relative contribution from the release of antibiotics, metals, biocides, and antibiotic resistance genes (ARGs) to the emergence, maintenance and spread of AMR in the environment?
(3) How well do current technologies and approaches limit AMR in the environment?
(4) If reducing AMR needs to be reflected within our regulatory framework, how could this best be tackled?
(5) Is there evidence to suggest that expansion of existing regulation/control measures for pollutants to AMR will translate into a decline in AMR in the environment?

Here, we review the pertinent issues that lie at the root of the aforementioned questions, such as: What are the drivers and pathways of AMR? And, How do these relate to the normal work, powers and duties of environmental regulators? The discussion is then turned to: What are the knowledge gaps that hinder the delivery of environmental protection from AMR? Finally, we offer several thought experiments for how different mitigation strategies might proceed in the light of a holistic understanding of AMR drivers and pathways. It is the intention that this review would be a catalyst for future discussions among scientists, policymakers, clinicians, veterinarians, and regulators. It is also our expectation that this review will stimulate a more systematic review and meta-analysis of the literature to further examine and critique the evidence base and the changing state of our knowledge gaps.

## Background of Global, Regional and National AMR Action Plans

**FIGURE 1:**
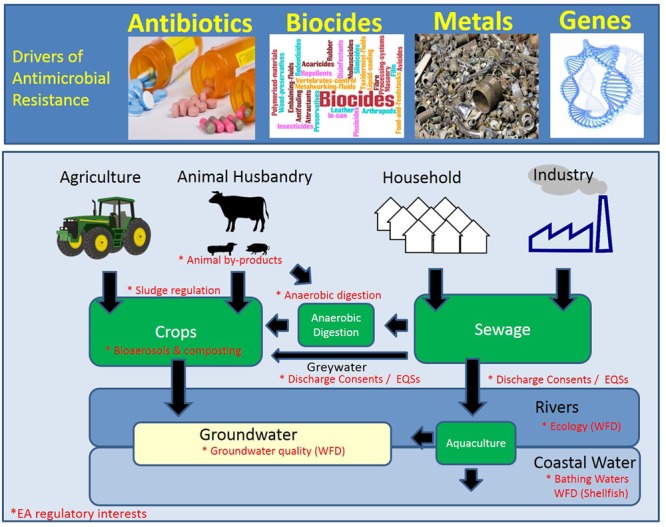
**Schematic of the hot-spots and drivers of antimicrobial resistance (AMR).** The environmental compartments that are currently monitored or regulated by the Environment Agency (EA; England) are denoted by an asterisk in red. WFD, Water Framework Directive.

The World Health Organisation (WHO) and its Global Action Plan broadly outlines five strategic objectives to tackle AMR: (1) to improve awareness and understanding of AMR; (2) to strengthen knowledge through surveillance and research; (3) to reduce the incidence of infection; (4) to optimize the use of antimicrobial agents; and (5) to ensure sustainable investment in countering AMR (World Health Organisation, 2015). The WHO highlights the role of the environment in Objective 4 of the Action Plan: “[To] Develop standards and guidance...for the presence of antimicrobial agents and their residues in the environment, especially in water, wastewater and food (including aquatic and terrestrial animal feed).” A similar action plan was published by the European Commission (2011). However, the only mention of the role of the environment was in ‘Action Number 8,’ which details the need to “Initiate cooperation on reduction of the environmental pollution by antimicrobial medicines particularly from production facilities.” The highlighting of pharmaceutical manufacturing is echoed in the O’Neill AMR Review, discussed below. Although rivers that receive eﬄuent from drug manufacturers have been shown to be a very important local issue with global implications (Larsson, 2014), it will be shown in subsequent sections to be only one pathway among several (see **Figure 1**). Moreover, it will be shown that antibiotics are only one of the many drivers of AMR in the environment.

The final Review on Antimicrobial Resistance entitled “Tackling drug-resistant infections globally: Final report and recommendations” (O’Neill and The Review on Antimicrobial Resistance, 2016) highlighted the need to reduce environmental pollution of antibiotics in much the same way as have done the WHO and EC AMR Action Plans. In brief, it highlights three pathways: (1) animal waste, (2) human waste, and (3) manufacturing waste. In every case the focus was on antibiotics, without any mention of other resistance-driving chemicals. It also prioritizes two pathways: hospital eﬄuent and pharmaceutical manufacturing plants. It is difficult to argue against improving these two pathways, as they are both highly relevant at a spatially resolved scale of analysis, however, these inputs are relatively small in number as compared to the overwhelming input of all resistance-driving chemicals from all the sources (see **Figure 1**). Moreover, it is unproven that WWTPs receiving eﬄuent from hospitals discharge significantly more resistance-driving chemicals into the environment, with measurably greater impact, than other similarly sized WWTPs. Although hospitals are an easy target and in many ways a tractable target, it does not necessarily make them the highest priority for reducing the prevalence of AMR in the environment. It is argued that the vision for these action plans is not sufficiently holistic to safeguard our natural environment. To appreciate these critiques, one must first understand what drives AMR in the environment.

## Fundamental Questions of AMR in the Environment: What are the Drivers and Pathways of AMR in the Environment?

Broadly speaking, antibiotic use in humans and animals carries an inherent risk of selecting for ARGs. These ARGs are often, if not always, found in bacteria with other genes promoting resistance to other potentially harmful chemicals (Alekshun and Levy, 1999; Levy, 2002; Enault et al., 2016; Subirats et al., 2016). It is most helpful to see these genes as ‘resistance genes’ (not ARGs), because antibiotics are not the only chemicals that select for resistance genes. It is an unfortunate historical convention to label all these genes antimicrobial resistance genes (ARGs), when in fact they can potentially confer resistance to many more chemicals.

A review of the literature highlights three well-characterized classes of resistance-driving chemicals: (1) antimicrobials, of which there are four subclasses, antibiotics, antifungals, antivirals, and antiparasitics; (2) heavy metals; (3) biocides (i.e., disinfectants and surfactants). However, many other chemicals, natural [e.g., plant-derived (Friedman, 2015)], and xenobiotic [e.g., solvents such as octanol, hexane and toluene (Ramos et al., 2002; Fernandes, 2003)], are also known to select for resistance genes. The prevalence of resistance genes in the environment are the result of a complex combination of factors, that reflect a dynamic balance of fitness costs and benefits: costs of carrying the ARG in the context of the host genome and environment (Maher et al., 2012; Roux et al., 2015); relative to the severity and frequency of hazard (Gullberg et al., 2011, 2014); relative to some physical environmental factors, such as temperature (Gifford et al., 2016) and microbial ecology (Amini et al., 2011), among others.

Co-selection of genes that confer resistance to chemical hazards [solvents (Fernandes, 2003), biocides (Pal et al., 2015; Wales and Davies, 2015; Wesgate et al., 2016), antibiotics (Levy, 2002; Blanco et al., 2016), and metals (Percival et al., 2005; Pal et al., 2015)] is a potentially ecologically and clinically important phenomenon. Co-selection is achieved in two ways: (1) co-resistance, whereby selection for one gene fosters the maintenance of another resistance gene, one that does not necessarily offer a selective advantage to the chemical in question (Johnson et al., 2016); and (2) cross-resistance, whereby one resistance gene can offer protection from multiple toxic chemicals (Curiao et al., 2016). Co-resistance is analogous to bringing a toolbox to a worksite; one might only need one or two tools from the toolbox at any one time, but there are many tools ‘fortuitously’ available for use should the need arise. The genomic architecture (e.g., ARGs found within: plasmids, transposons, and integrons) is the toolbox and the resistance genes that are co-located in the genome are the tools. Cross-resistance is analogous to having a tool that is capable of multiple functions, such as a claw hammer, which can hammer in as well as extract a nail, thereby offering multiple functions from the same tool. Eﬄux pumps can often provide cross-resistance to multiple chemicals.

The literature describes three major pathways for resistance-driving chemicals into the environment (see **Figure 1**):

(1) Municipal and industrial wastewater;
(2) Land spreading of animal manure and sewage sludge; and
(3) Aquaculture.

Additional pathways will be discussed (e.g., aerosols and mining), however, they were not included in **Figure 1** as it was felt that the evidence base for their importance needs further strengthening.

## Drivers of Resistance: Antibiotics

### Scale of Human Antibiotic Use

In 2013, the total measured consumption of antibiotics in England was 27.4 DDD per 1000 inhabitants per day [general practice 79%, hospital 15% and other community consumption (predominantly dentists) 6%], in line with the median across Europe in 2011 of 21.3 median DDD per 1000 inhabitants per day (Public Health England, 2014), where the DDD is the assumed average maintenance dose per day for a drug used for its main indication in adults. A total of 66 different antibiotics were prescribed, with the top 15 antibiotics in general practice and hospitals accounting for 98 and 88% of consumption, respectively (Public Health England, 2014).

In 2010, India was the largest consumer of antibiotics when assessing total tonnage, however, their per capita usage (7.5 units per capita), was low by comparison to Australia and New Zealand which recorded among the highest usage rates of 87 and 70 units per capita, respectively (Van Boeckel et al., 2014). An antibiotic unit was defined in this study as the number of doses (i.e., pill, capsule, or ampoule) sold (Van Boeckel et al., 2014). China was the second largest consumer of antibiotics, globally when assessing total tonnage, but similar to India, recorded relatively low antibiotic usage per capita (7.5 units).

Heterogeneity in antibiotic use is reproduced at seemingly every geographical scale. For example, nested within the larger global differences are differences between countries within smaller regions, e.g., Europe, where nearly 300% more antibiotics were used per capita in Turkey than Armenia (Versporten et al., 2014). Within Europe, the highest defined daily dose (DDD) of antibiotics (outpatient) per capita is France (32 DDD) and Greece (30 DDD), with the UK (15 DDD) exceeding that of the lowest use European country, The Netherlands, by 33% (Goossens et al., 2005). Differences between countries is often attributed to the ease at which one can self-medicate, with eastern and southern European countries (e.g., Bulgaria, Cyprus, Greece, Lithuania, Romania, and Spain), demonstrating greater access to antibiotics without a prescription, thereby increasing use and misuse (Safrany and Monnet, 2012). In general, per capita antibiotic use across Europe is lowest in northern regions, moderate in eastern regions and highest in southern regions (Goossens et al., 2005).

In addition, to there being large differences between countries within regions, there are significant differences in antibiotic use within countries. The highest combined general practice and hospital antibiotic consumption was in Merseyside, with similar levels reported in Southern Europe with 30.4 DDD per 1,000 inhabitants per day, over 30% higher than Thames Valley with the lowest consumption, (22.8 DDD per 1,000 inhabitants per day) (Public Health England, 2014). Durham, an area in the northeast of England reported 40% more antibiotic prescriptions in general practice than in London (southeast England) (Public Health England, 2014). The ESPAUR Report 2014 notes that areas within the UK that report high antibiotic prescription rates also reported higher antibiotic resistance rates; a finding the authors acknowledged necessitates further examination. It remains unexplored as to whether these regional variations in antibiotic prescriptions translate into differences in risk to antibiotic resistance selection and maintenance in the environment. The evidence of heterogeneous antibiotic use across large and small scales likely has multiple causes and as such, AMR Action Plans need to be sufficiently holistic to address the diversity of aetiologies.

### Scale of Animal Antibiotic Use

According to The State of the World’s Antibiotics 2015, two-thirds of all of the antibiotics produced globally each year (65,000 tons of 100,000 tons) are used in animal husbandry (Gelband et al., 2015). The variability between countries in veterinary antimicrobial use in food-producing animals just within the high income countries can be significant. Sales of veterinary antimicrobial agents among the 26 EU countries in 2013 expressed as mg of antibiotic per population correction unit (PCU) places Norway as having the lowest mg/PCU at: 3.7 mg/PCU, with the UK at 62.1 mg/PCU. PCU is a standard unit of measure that takes into account the number of animals in a country and their average weight at the point they are most likely to be treated, providing an estimate of total kg of food producing animal in a country. At the upper end of veterinary antimicrobial use in the EU, was Italy (301.6 mg/PCU), Spain (317.1 mg/PCU), and Cyprus (425.8 mg/PCU) (European Medicines Agency, 2015). The majority of these sales are for tetracycline and penicillin antibiotic classes, composing between 6–56 and 11–61% of the total antibiotics sold in each country for food-producing animals, respectively (European Medicines Agency, 2015). In combination with sulfonamides, these three classes of antibiotic account for 71% of the total sales in these 26 European Union (EU)/European Economic Area (EEA) countries in 2013.

In 2013, the total antibiotic sales for therapeutic use in animals, was 420 tons, approximately 44% of the total antibiotic usage in the UK (Public Health England, 2015). The number of livestock in the UK in 2013 was: cattle (9.8 m), pigs (4.9 m), sheep (32.8 m), chicken (162.6 m) (Veterinary Medicines Directorate, 2014). The milligrams of active ingredient of critically important antibiotics (CIA) sold for food producing animals per PCU for 2013 was: cattle (8 mg/pcu), pigs and poultry (172 mg/pcu) (Veterinary Medicines Directorate, 2014). Tetracyclines were the most frequently used antibiotics in animals (43.5%), followed by penicillins (21.7%), mirroring the use patterns in humans where it was reversed, penicillins (64%) followed by tetracyclines (10%) (Public Health England, 2015). The estimate that roughly 44% of the antibiotics used in the UK are for veterinary use, is much lower than estimates for other countries, such as the U.S., where >70% of antibiotics are estimated to be used in livestock (O’Neill and The Review on Antimicrobial Resistance, 2015).

Antibiotics are important for maintaining animal health and welfare. Antibiotics are often dispensed to treat or prevent infections in herds/flocks (Gelband et al., 2015). Antibiotics are often added to the animal’s water or food as a pragmatic solution to the fact that they are reared in groups/flocks making it difficult to isolate and treat only the infected. In addition, the effort to isolate animals could be stressful to the animal and sometimes dangerous to the veterinarian who administers the antibiotic.

The use of antibiotics for growth promotion has been banned in Europe and is in decline in North America. Major food suppliers such as Tyson (Tyson Fresh Meat Inc., 2014), McDonalds (McDonalds Corporation, 2015), Chick-fil-A (Chick-fil-A, Inc., 2014), Subway (Subway Ip, Inc., 2016), and Taco Bell (Taco Bell Corp, 2016) are slowly phasing in new commitments to greatly restrict antibiotic use in food production, largely in poultry.

### Relevant Pathways for Antibiotics

#### Municipal and Industrial Wastewater

A large fraction of the antibiotics consumed by humans are excreted in the urine and feces in their biologically active form (Singer et al., 2011; Zhang et al., 2015; Verlicchi and Zambello, 2016). The antibiotics excreted by humans will enter WWTPs, with one of three fates: (1) biodegradation (Chen et al., 2015); (2) absorption to sewage sludge (Li and Zhang, 2010; Ahmed et al., 2015); or (3) exit in the eﬄuent unchanged (Rivera-Utrilla et al., 2013; Luo et al., 2014). Biologically active metabolites of the antibiotic which can be generated in the wastewater and in the wider environment are not being considered in this review, though they are potentially ecologically relevant (Gros et al., 2013).

The persistence of an antibiotic in a WWTP is a function of: (1) influent composition (industry, municipal) (Gardner et al., 2013; Larsson, 2014); (2) salinity; (3) temperature; (4) nature of WWTP (e.g., trickling bed filter, activated sludge, and membrane bioreactor); and (5) hydraulic retention time. A survey of 16 UK WWTPs revealed the presence of erythromycin, ofloxacin, and oxytetracycline (the only three for which they authors assayed) in each of the WWTPs (Gardner et al., 2013). The median concentration of erythromycin was 2.0 μg/L ± 0.8 standard deviation [(SD; 42% coefficient of variation (CoV)]. The median concentration of ofloxacin had was 0.18 μg/L ± 0.33 SD (178% CoV), while oxytetracycline had a median concentration of 3.6 ± 2.5 SD (70% CoI) (Gardner et al., 2013). The CoV for these antibiotics, ranged from 42 to 178% reinforcing the fact that sewage influent composition has a high degree of variability. The affinity of oxtetracycline to bind to sludge led to relatively high concentrations in the sludge as compared to other pharmaceuticals in the influent (1.15–43 mg/kg) (Gardner et al., 2013). The preferential removal of some antibiotics [e.g., sulfonamides (Yang et al., 2012), ciprofloxacin (Polesel et al., 2015)] into the sludge suggests that the risks from sludge application to land are likely to be different from the risks from discharging sewage eﬄuent into rivers. The authors also reported enhanced removal of pharmaceuticals where there was iron dosing at the primary treatment stage of a trickling filter WWTP (Gardner et al., 2013). Specifically, they reported an average loss of erythromycin, ofloxacin and oxytetracycline of 20, 74, and 51%, respectively, after iron-dosing (used to remove phosphorus), as compared to undosed primary treatment of -11, 19, and -4%, respectively. Concentrations of oxytetracycline within a river catchment receiving WWTP eﬄuent varied 14-fold across nine WWTP sampling days, from 9.4 to 137 g/d (normalized to flow) (Comber et al., 2015), illustrating the variability in antibiotic consumption and the extent to which environmental exposures to antibiotics can fluctuate.

#### Greywater, Reclaimed and Black Water

The global water footprint in the period 1996–2005 was 9087 Gm^3^ year^-1^, of which 15% was categorized as gray water (UNESCO-IHE, 2011). Greywater is defined as water originating from the mains potable water supply that has been used for bathing or washing dishes or laundering clothes, excluding toilet water (Finley et al., 2009). Reclaimed water is typically water that originates from WWTP eﬄuent that has undergone additional treatment to ensure its safe use in a variety of applications, including irrigation and toilet flushing (Kinney et al., 2006). Blackwater is recycled, treated sewage eﬄuent (Otterpohl et al., 2004). The use of reclaimed water for irrigation purposes would be subject to Discharge Consent by the Environment Agency and would need to comply with British Standards (e.g., BS 8595:2013 Code of practice for the selection of water reuse systems; BS 8525-1:2010 and BS 8525-2:2011 Greywater systems. Domestic greywater treatment equipment. Requirements and test methods). While sewage wastewater in the U.S. must meet treatment guidelines set by individual states before used for irrigation, regulations do not require wastewaters originating from animal feeding lots to be treated before land application (Dodgen and Zheng, 2016).

Although the use of reclaimed water is relatively new in the UK, it’s a practice that is well-established in hot, dry climates where water pressures have been more acutely sustained (Hamilton et al., 2005). The prospect of less reliable water sources into the future, as a result of a changing climate, might make this source of water more important in the UK (Prudhomme et al., 2012). Reclaimed water is currently used to sprinkler irrigate crops (e.g., lettuce, carrots, and green beans), golf courses, and landscapes (Kinney et al., 2006; Calderón-Preciado et al., 2013; Thanner et al., 2016), and as such is being introduced into soil habitats that might have previously been unexposed to significant quantities of ARGs or resistance-driving chemicals (Fahrenfeld et al., 2013; Han et al., 2016). The amplification of antibiotic resistant bacteria within distribution system for reclaimed water is poorly understood, but poses a potential risk to humans and the environment (Fahrenfeld et al., 2013). Both treated sewage wastewater and animal feeding operations wastewater can have high levels of dissolved organic matter and nutrients as well as a highly variable microbial community activity and composition (Dodgen and Zheng, 2016). The interaction between the microbial community, the antibiotics and the dissolved organic matter can greatly impact the fate of the antibiotics in the soil environment through a combination of biodegradation and adsorption (Dodgen and Zheng, 2016).

#### Veterinary and Livestock

As in humans, when animals consume antibiotics as much as 30 to 90% is released into the manure and urine (Sarmah et al., 2006; Berendsen et al., 2015). Animal excreta has been shown to contaminate the environment with antibiotic resistant bacteria and antibiotics (Udikovic-Kolic et al., 2014; Wichmann et al., 2014). This phenomenon was recently demonstrated in a survey of feces from 20 commercial swine and 20 cattle farms in The Netherlands. The study reported antibiotics in 55% of the swine feces from 80% of the swine farms and 75% of the calf feces from 95% of the cattle farms (Berendsen et al., 2015). Among the antibiotics recovered, oxytetracycline, doxycycline, and sulfadiazine were the most frequent, followed by tetracycline, flumequine, lincomycin, and tylosin. Over one-third of the feces samples contained more than one antibiotic; as much as three different antibiotics in swine feces and eight different antibiotics in cattle feces. The authors concluded that the sum of the concentrations of different antibiotics within a sample exceeded concentrations needed to select for antibiotic resistance, e.g., the minimum selective concentration (MSC).

The observed transmission of resistance genes through contaminated cow and calf bedding and soil (Call et al., 2013; Kyselková et al., 2015; Liu et al., 2016), highlights the importance of biosecurity and the need to separate animals being treated for infection from the herd (where feasible), and not reusing bedding from infected and treated animals (Mitchell et al., 2015). Intramuscular treatment of healthy calves with ceftiofur or florfenicol, as per manufacturer’s recommendations, was shown to be sufficient to establish a reservoir of drug-resistant *Escherichia coli* in the feces, soil and bedding of treated animals (Liu et al., 2016). The dairy calves were housed individually in indoor pens with composted bedding material. Elevated levels of drug-resistant *E. coli* were maintained in the feces (collected directly from the rectum) and the bedding for a month. Treatment of the calves with oxytetracycline resulted in no significant effect–a result the authors attribute to the small trial size, but similar to a previous study comparing the differential effects of antibiotic administration route on antibiotic resistance generation in the microbiome (Zhang et al., 2013). The authors also introduced a multi-drug resistant marked *E. co*li into the bedding of calves and found a significant increase in the *E. coli* population in the bedding of calves treated with ceftiofur as compared to the untreated control calves (Liu et al., 2016). Bedding-to-animal transmission was confirmed when calves were found to be shedding the multi-drug resistant marked *E. coli* in their feces. The authors also found co-selection of florfenicol-resistant *E. coli* after treatment of animals with ceftiofur and vice versa. This study exemplifies many of the factors that make control of AMR in a veterinary setting difficult: (1) persistence and shedding of the drug into the environment in urine and feces; (2) selection of antibiotic resistant organisms in the environment; and (3) transmission of drug-resistant microbes acquired from the environment (Liu et al., 2016).

The transmission of antibiotic resistant bacteria and genes from animals to humans has been demonstrated in the literature (Khanna et al., 2008; Wulf et al., 2008; Smith et al., 2013). On-farm transmission of AMR has been characterized in the literature for a wide range of animals: pigs (Crombé et al., 2013; Novais et al., 2013), cows (Wichmann et al., 2014), and insects (Zurek and Ghosh, 2014; Usui et al., 2015; Hammer et al., 2016). A recent review of the academic literature that address the issue of antibiotic use in agriculture suggests that only seven studies (five percent) argued that there was no link between antibiotic consumption in animals and resistance in humans, while 100 (72%) found evidence of a link (O’Neill and The Review on Antimicrobial Resistance, 2015). The degree to which this transmission from animals to humans, and vice-versa, is of great interest and has significant implications for public and animal health (Weir et al., 2012; Woolhouse et al., 2015; Klous et al., 2016). The relevance of theses pathways to the environment will be discussed, as this is the main remit and interest of environmental regulators.

#### Land Application of Manure and Sludge

Biological treatment of wastewater generates sludge or ‘biosolids,’ a waste material of the WWTP that is high in undegraded protein, oils and fats as well as microorganisms and undegraded pharmaceuticals, the latter of which can reflect hundreds of different chemicals from sub-ng/L to >10 μg/L (Fick et al., 2010; McClellan and Halden, 2010). An estimated 37% of biosolids are land applied in Europe, equating to approximately 2.39 × 10^6^ dry tons per year (Kinney et al., 2008). In 2008, approximately 1.4 million tons of dry sludge solids were produced in England and Wales, 77% of which was spread on land for agricultural benefit (Water UK, 2010). Biosolids make up less than 5% of the total organic material going to agricultural land (Water UK, 2010). All biosolids used on agricultural land in the UK are applied in accordance with The Sludge (Use in Agriculture) Regulations and The Code of Practice for the Agricultural Use of Sewage Sludge. The regulations are primarily concerned with the impact from metals contained in the sludge on the receiving soil and adjacent water bodies (ground and surface), however, they generically address any component of the sludge that negatively impacts the yield of crops or puts animal or human health at risk either directly or through the food chain (Defra, 2006).

The antibiotics that are more frequently found in sludge are those that are generally less water soluble, e.g., norfloxacin, ofloxacin, ciprofloxacin, trimethoprim, sulfamethoxazole, and doxycycline (McClellan and Halden, 2010; Clarke and Smith, 2011). The concentration of antibiotics within sludge and manure will vary greatly depending on the origin of the influent, treatment conditions, partitioning properties of the antibiotics, and environmental conditions (Hörsing et al., 2011; Chen et al., 2013; Li et al., 2013). Two biocides, triclocarban and triclosan were the most abundant analytes in a large survey of pharmaceuticals and personal care products in sewage sludge in the U.S. They reached concentrations as high as: 48.1 and 19.7 mg kg^-1^ (dw), respectively, with mean concentrations of: 36 ± 8 and 12.6 ± 3.8 mg kg^-1^ (dw). The second most abundant class of chemicals was antibiotics, in order of decreasing concentration: ciprofloxacin > ofloxacin > 4-epitetracycline > tetracycline > minocycline > doxycycline > azithromycin, ranging between: 6.8 ± 2.3 and 0.8 ± 0.2 mg kg^-1^ dw (McClellan and Halden, 2010). Notably, the biocides and antibiotics explored by McClellan and Halden (2010) were typical of the small subset of antibiotic and biocides with a high affinity for sorption to sewage sludge (Li and Zhang, 2010).

Toxicological and AMR selection risks cannot be easily predicted from solely knowing the concentration of antibiotics, metals and biocides owing to significant uncertainties in bioavailability (Jechalke et al., 2014; Aga et al., 2016). Fluoroquinolones have been shown to be relatively biounavailable in some sludge amended soils, which was not correlated with any significant change to the ecosystem services: nitrification and denitrification; however, community structure and other ecosystem services might have been impacted, but were not examined (Rosendahl et al., 2012). Similarly, soil fauna, such as earthworms, springtails, and enchytraeids exposed to environmentally relevant concentrations of antibiotics in soil showed no significant reduction in reproduction (Baguer et al., 2000). Lowering of antibiotic, biocide and metal bioavailability in sludge will often decrease the exposure and the toxic response in mesofauna. Similarly, by lowering the bioavailability of these chemicals it is likely that the hazard to microorganisms will decrease, which might result in a reduction in the selective pressure (Jechalke et al., 2014; Dodgen and Zheng, 2016). It remains undetermined the extent to which bioavailability controls resistance gene selection in the environment–an issue that emerges as a significant knowledge gap.

## Ecological Relevance of Antibiotic Concentrations

The well-established minimum inhibitory concentration (MIC) in clinical settings refers to the concentrations needed to inhibit the growth of, or kill, a target pathogen. The concentration at which a resistance genes offers a selective advantage to its ‘host,’ will be below the MIC, otherwise the microorganism would not survive. The MSC is a useful term that refers to the minimal concentration of chemical required to provide a selective advantage to a microorganisms carrying the resistance gene relative to the same bacterium that is sensitive to the chemical, i.e., not containing the resistance gene (Gullberg et al., 2011; Hughes and Andersson, 2012; Lundström et al., 2016). The MSC is a theoretical threshold that can be carefully determined in the laboratory for any microorganism and chemical pair (Liu et al., 2011), but takes on a different meaning when applied to more realistic scenarios of multiple species and multiple chemicals. Efforts to determine MSCs for more complex systems are ongoing, and as a result of their more realistic conditions might offer greater insights into the ecological relevance of low concentrations of antibiotics in the environment (Yergeau et al., 2010, 2012). The MSC has been shown to be sensitive to a range of biological and physico-chemical factors (Gullberg et al., 2014). Heterogeneity in the concentration of an antibiotic through differential partitioning into soil pores, sediment, biofilms, organic matter, are thought to play a significant role in allowing for the co-location of antibiotic resistant and sensitive microorganisms (Baquero and Negri, 1997; Hughes and Andersson, 2012). Heterogeneity in the exposure of microorganisms to weakly selective sub-lethal concentrations of antibiotic within a matrix (e.g., soil, sediment, leaf, suspended particulate, and biofilms) allows for the selection and persistence of the more common, frequent small-effect mutations. The proximal location of microorganisms to higher or lower concentrations of these resistance-driving chemicals further aids in the establishment of these accumulated small mutations in the population (Hughes and Andersson, 2012; Chait et al., 2016).

## Drivers of Resistance: Biocides

Biocides are disinfectants that are commonly used in hospitals, cosmetics, household cleaning products, wipes, and furniture preservatives, farmyards for purposes such as: wheel and foot washes and a range of industrial processes, including the control of fouling and souring of pipes including oil wells (e.g., hydraulic fracturing) (Kahrilas et al., 2015). Some common biocides are: ethanol, formaldehyde, chlorhexidine, triclosan, and quaterium ammonium compounds [QACs, e.g., BC also known as alkyldimethyl-benzyl-ammonium chloride (ADBAC), stearalkonium chloride, isothiazolium-benzalkonium chloride, cetrimonium chloride/bromide (cetrimide), cetylpyridinium chloride, alkyl amino alkyl glycines and didecyldimethylammonium chloride (DDAC)] (Russell, 2003; Buffet-Bataillon et al., 2012). In Europe, biocide marketing, use and disposal is regulated under the Biocidal Products Regulation (EU) 528/2012 (European Commission, 2012). The legislation also controls their use in the veterinary setting. The global market for biocides grew by 40% between 1992 and 2007 (Sattar et al., 2007).

The MIC for six different biocides has been shown to range from 0.4 to >1000 mg/L across 16 different bacteria (e.g., *Pseudomonas aeruginosa, E. coli, Staphylococcus aureus, Citrobacter* sp.). Triclosan, along with other biocides, such as chlorhexidine and QACs, have been shown to select for antibiotic resistance (European Commission, 2009; Bridier et al., 2011; Carey and McNamara, 2014; Webber et al., 2015; Buffet-Bataillon et al., 2016). In much the same way that sub-lethal concentrations of antibiotic can select for ARGs, sub-lethal concentrations of biocides have also been shown to select for common mutations that confer clinically relevant antibiotic resistance (Webber et al., 2015). As previously discussed, some resistance mechanisms are common to both biocides and antibiotics, allowing for co-selection of ARGs (McBain et al., 2002; Levy, 2002; Russell, 2003). Co-resistance of *S. aureus* to the biocide benzalkonium chloride and the antibiotic oxacillin, affording the resistant strain eightfold higher tolerance to the antibiotic than its sensitive wild-type strain (Yamamoto et al., 1988). The frequent co-location of these genes on plasmids (a small DNA molecule within bacteria that are physically separate from chromosomal DNA and can replicate independently) with ARGs affords them greater resilience and mobility, allowing for their rapid spread through microbial communities and pathogens (Gaze et al., 2005; Pal et al., 2015).

The ecological relevance of biocides will need to be determined to effectively risk rank resistance-driving chemicals for mitigation–an issue that will be discussed in greater detail in following sections. The Scientific Committee on Emerging and Newly Identified Health Risks on behalf of the European Commission highlighted the potential consequences to the environment and human health from widespread biocide use in a recent report entitled “Assessment of the Antibiotic Resistance Effects of Biocides” where they call for: (1) a better understanding of the quantity of biocides and their residues in the environment and its effects on AMR selection; (2) methodologies for assessing impact on AMR; and (3) studies characterizing AMR and co-selection (European Commission, 2009).

### Relevant Pathways for Biocides

The route into the environment for biocides is similar to antibiotics, most notably WWTPs. Improper disposal of biocides through dilution and discharge to WWTPs can also increase the load of biocides entering the environment, while also increasing the likelihood of generating resistance.

## Drivers of Resistance: Metals

Major urban inputs of heavy metals to WWTPs come from household eﬄuent, drainage water, business eﬄuent (e.g., car washes, dental uses, other enterprises), atmospheric deposition, and traffic-related emissions (vehicle exhaust, brake linings, tires, asphalt wear, gasoline/oil leakage) (Karvelas et al., 2003). Metal nanoparticles (e.g., titanium, copper, and silver) have become widely used in food, textiles, household, industrial and hospital products, and disinfectants (Selck et al., 2016). Metals such as Pb, Cu, Zn, Cd, and As have been used as animal growth promoters and nutritional supplements (European Commission, and Health and Consumer Protection Directorate-General, 2003), fertilizers, pesticides, and fungicides in aquaculture and agriculture (Pal et al., 2014).

The annual heavy metal inputs to agricultural land in England and Wales for the year 2000 was substantially higher for livestock manures than for any other soil input (Nicholson et al., 2003). Sources of heavy metal input to agricultural soil, in decreasing mass (g/ha/yr), was: livestock manure (11,312), sludge (4,557), paper sludge (1,380), fertilizer [89.7; including: nitrogen (2.2), phosphorus (34), potash (0.5), lime (53)], atmosphere (221) and irrigation water (39) (Nicholson et al., 2003). The majority of heavy metals originating from animals can be found in the feed, with on average: 150–2920 mg Zn/kg dry matter and 18–217 mg Cu/kg dm in pig feeds–values of which depend on the age of the pigs (Nicholson et al., 1999). Poultry feeds contained a wider range, with: 28–4030 mg Zn/kg dm and 5–234 mg Cu/kg dm, again with variation in the age and breed of chicken (Nicholson et al., 1999). While concentrations of Zn and Cu in dairy and beef cattle feeds were much lower than in pig and poultry feeds. Dairy and beef cattle feed (dairy cake/nuts) contained 130–190 mg Zn (dm) and 35–40 mg Cu/kg (dm) (Nicholson et al., 1999). Supplements contained a mean of 2900 mg Zn/kg and 1500 mg Cu/kg (dm), while also containing higher levels of Ni, Pb, Cd, As, and Cr than other feed components (Nicholson et al., 1999).

Metal and antibiotic resistance can share structural and function resistance systems, such as: (1) reduced membrane permeability (metals: As, Cu, Zn, Mn, Co, Ag and antibiotics: ciprofloxacin, tetracycline, chloramphenicol and β-lactams); (2) drug and metal alteration (metals: As, Hg and antibiotics: β-lactams, chloramphenicol); (3) drug and metal eﬄux (metals: Cu, Co, Zn, Cd, Ni, As and antibiotics: tetracyclines, chloramphenicol, β-lactams); (4) alteration of cellular targets (metals: Hg, Zn, Cu and antibiotics: ciprofloxacin, β-lactams, trimethoprim, rifampicin); and (5) drug and metal sequestration (metals: Zn, Cd, Cu and antibiotics: coumermycin A) (Baker-Austin et al., 2006).

The toxicity of metals to microorganisms directly relates to the affinity of the metal to thiol compounds such as glutathione within the cell, in order of increasing ‘toxicity’/S-affinity: Mn < Co < Zn < Ni < Cd < Pb << Cu << Ag < Hg (Nies, 2003). A log change in sulfur affinity brings a log decrease in the quantity of metal required to inhibit growth, i.e., increase in toxicity (Nies, 2003). This series of metals with increasing toxicity compares in an intriguing manner with a database of antibacterial biocide-and metal-resistance genes, BacMet (Pal et al., 2014). Of the 20 metal resistance genes for which there is experimental evidence of their function, copper genes are the most abundant, followed by, in decreasing order of their abundance in the database, with those metals that are non-essential for life in braces: Cu, Zn, Ni, (Cd), Co, Fe, Mn, (As), (Ag), (Te), (Hg), (Pb), Mn, (Cr), (Au), (Ga), (Sb), (V), Se, (Bi) (Pal et al., 2014). A rationale for this distribution will likely relate to a balance of fitness costs and benefits, which includes: (1) Discrepancies between the toxicity ranking and prevalence of metal-resistance genes in the BacMet database might be explained by: incomplete annotation of the function of some genes; non-exhaustive surveys of gene prevalence in the lab and environment; and non-uniform exposure and selection of metals and metal-resistance genes in the environment.

Considerable attention has been given to understanding the speciation of metals for predicting their mobility and toxicity in soils, sediment and aquatic system (Olaniran et al., 2013; Tipping and Lofts, 2013; Ardestani et al., 2015). However, it remains largely unexplored as to the relationship between metal bioavailability, speciation and resistance gene selection. The desire to limit resistance selection in soils might necessitate rethinking the environmental thresholds set for manure and sludge land spreading as the combined effects of antibiotics, biocides and metals might exceed the MSC for some shared resistance mechanisms.

Biological wastewater treatment processes have been shown to be disrupted as a result of the toxic effects of heavy metals in the influent (Giller et al., 1998; Smith, 2009), such as: nitrification (Braam and Klapwijk, 1981), denitrification (Waara, 1992), and organics removal (Ajmal et al., 1983). The toxicity of heavy metals in wastewater is sensitive to the species and concentration of metal, pH, sludge concentration, wastewater pollution load and solubility of the metal ions, among other factors (Chipasa, 2003). Bacteria carrying metal resistance genes have been shown to more frequently carry ARGs as compared to those bacteria without metal resistance genes, and these genes can often be found on plasmids (Pal et al., 2015; Di Cesare et al., 2016). It is common for a microbe to have both ARGs and metal resistance genes, however, a study of 4582 plasmids revealed that in only 5% of the cases are they both found on the same plasmid (Pal et al., 2015). It remains unclear whether the public database of plasmids from which this study was based offers a representative view of plasmid ecology; future targeted and controlled studies will help to support or challenge this important observation.

### Relevant Pathways for Metals

Metals have similar pathways to that of biocides and antimicrobials, however, the relative proportion of each source is likely to differ depending on the prevalence of industry, agriculture (i.e., pesticides and biosolids), or waste water/stormwater. Rivers downstream of urban areas, where stormwater runoff is acute, and regions that have experienced mining, are often impacted with elevated concentrations of metals (Johnston et al., 2008). It remains unclear as to the ecological relevance of these diffuse and point sources of metals to the selection for resistance in the environment.

## Drivers of Resistance: Antibiotic Resistance Genes

The chronic release of antibiotics into WWTPs is concomitant with the release of resistance genes. Resistance genes in wastewater originate from the gastrointestinal tract of, primarily, humans (Hu et al., 2013; Chang et al., 2015; Newton et al., 2015). The co-location of antibiotics and ARGs in WWTPs can (and does) select for novel combinations of AMR that can be shared between microorganisms by horizontal gene transfer (HGT) on mobile genetic elements (MGEs), such as plasmids, thereby increasing the prevalence and combination of multiple drug resistance in the microbial community (Szczepanowski et al., 2005; Xu et al., 2015). The competitive and chemically challenging environment of a sewage works offers favorable conditions for the amplification of existing resistance genes, creation of novel resistance genes or genomic assemblages (Zhang and Zhang, 2011).

Antibiotics can trigger the bacterial SOS response in which bacteria undergo a transient, genome-wide hypermutation that generates genetic variability in times of stress (Baharoglu and Mazel, 2014). The SOS response can also trigger HGT of ARGs, thereby providing a mechanism for the spread of ARGs from exposure to antibiotics (Beaber et al., 2004). Many additional drivers (e.g., chemical and environmental) of HGT have been identified (Hastings et al., 2004; Antonova and Hammer, 2011), including abiotic sources (Warnes et al., 2012; Kotnik, 2013). The relative importance of each of these drivers of HGT has yet to be assessed at an ecologically relevant scale.

Resistance can be intrinsic, which suggests it is universally found within the genome of the bacterial species, while acquired resistance suggests it could be acquired by means of new genetic material or through sporadic mutations of intrinsic genes (Hollenbeck and Rice, 2012). Intrinsic resistance is maintained by a species independent of any antibiotic selective pressure and, by definition, is not acquired by HGT (Cox and Wright, 2013). Antibiotic-producing environmental microorganisms, unlike clinical microorganisms, demonstrate a significant degree of intrinsic resistance that appears to be independent of the selective pressure–an attribute that has been suggested to be ancient and long predates clinical antibiotic uses (D’Costa et al., 2011; Cox and Wright, 2013). The risk of intrinsic resistances found in environmental microorganisms being transferred to pathogens is already apparent and of significant international concern (Forsberg et al., 2012; Cox and Wright, 2013; O’Neill and The Review on Antimicrobial Resistance, 2015).

Intrinsic resistance can be further enhanced through the modification or enhancement of intrinsic resistance, e.g., upregulation of cellular components that protect against the effects of the antibiotic [e.g., penicillin-binding protein 5 (PBP 5)] (Sifaoui et al., 2001), such modifications are then considered acquired resistance. A major mechanism for acquired resistance is through MGEs. MGEs can mobilize between cells through: (1) transformation, (2) conjugation and (3) transduction. Transformation is the acquisition of naked DNA from the environment (Davison, 1999; Gaze et al., 2013). Conjugation is the transfer of genes through direct contact between two bacteria (Lopatkin et al., 2016). Transduction is the transfer of genes from bacteriophage (viruses that infect bacteria) to bacteria during infection of a bacterial cell (Brown-Jaque et al., 2015; Calero-Cáceres and Muniesa, 2016). MGEs can also be distinguished by their ability to freely transmit from cell to cell (i.e., intracellular MGEs) and those that can only transmit from cell to cell after integrating into intracellular MGEs (Siguier et al., 2014). Intracellular MGE or transposable elements (TEs) include transposons (Tn), insertion sequences (ISs) and integrons (In) (Siguier et al., 2014). Transposons are made up of conjugative transposons and integrative and conjugative elements (ICEs) (Wozniak and Waldor, 2010). ICEs are genetic elements found in both Gram-positive and Gram-negative bacteria that are either self-transmissible or use mobile plasmids and viruses as vehicles for dissemination (Wozniak and Waldor, 2010). The relevance of all these MGEs is that many contain ‘cargo genes’ that can confer phenotypes such as antibiotic resistance to the receiving cell (Roberts and Mullany, 2011; Johnson and Grossman, 2015). It is the numerous ways for resistance genes to mobilize and transfer from cell to cell that makes the environmental resistome of human and animal relevance (Perry and Wright, 2013). It goes beyond the scope of this review to further detail the many different mechanisms of MGE, a number of excellent papers cited above and here are recommended (Frost et al., 2005; Wiedenbeck and Cohan, 2011; Perry and Wright, 2013).

## Relevance of AMR to Environmental Regulators?

There are a large number of activities or products that are currently monitored or regulated by the Environment Agency of England, which intersect with the pathways and drivers of AMR (**Table 1**). Equivalent legislation, if it exists, will differ by country, though many regulations are treated similarly across countries within the European Community with which England is aligned.

**Table 1:** Environment and activities or products monitored or regulated by the Environment Agency of England, which play a potentially important role in the spread and maintenance of AMR in the environment.

Environment	Intersection of Environment Agency with AMR
Wastewater treatment plant (WWTP)	^1^Discharge of treated eﬄuent (from industry and municipal sewage) to land, coast or rivers. ^1^Disposal of sewage sludge ^1,2,3^Disposal of anaerobic digestate
Agriculture	^1^Land spreading of manure, sewage sludge, and anaerobic digestate as fertilizer or soil conditioner. ^1^Bioaerosols from agriculture (pig and poultry farming) and composting.
Animal husbandry	^1^Disposal of animal by-products ^1,2^Disposal of animal slurry and manure ^1,2,3^Disposal of anaerobic digestate
River water quality	^1,4^Impact of sewage eﬄuent ^1,4^Impact of diffuse pollution from farm-yard, manure- and biosolid-amended agricultural soil and storm runoff ^1,4^Freshwater fish farm
Coastal and bathing waters	^1,4^Impact of farmyard runoff and sewage eﬄuent on bathing water quality, and shellfish bed water quality ^1,4^Impact of aquaculture on coastal water quality.
Groundwater quality	^1,5^Leaching of soil amendments (biosolids and manure) and chemical crop treatments

*Key regulations: ^1^Environmental Permitting (England and Wales) Regulations 2010 (Defra, 2013); ^2^Animal By-Products (Enforcement) (England) Regulations 2013 (Public Health England, 2013); ^3^Anaerobic digestate: PAS 110:2014 (BSI, 2014); ^4^Water Framework Directive 2000/60/EC (WFD) (European Commission, 2000); ^5^The Sludge (Use in Agriculture) Regulations 1989 (Public Health England and Wales, and Public Health Scotland, 1989).*

### WWTP Discharge

Human use antibiotics, biocides, metals and ARGs all enter STPs, however, they are discharged as a component of the: (1) eﬄuent, and (2) sludge (biosolids). The sludge can be composted or anaerobically digested and applied to land or incinerated. The proportion of antibiotic, metal, and biocide in the eﬄuent and sludge is highly variable depending on the STP catchment characteristics, presence of hospital wastewater, nature of STP and its operational parameters. WWTPs do not efficiently remove all ARGs that are subsequently released in treated eﬄuents (Yang et al., 2014). So the key question is whether these ARGs contribute to the increased prevalence of ARGs in the environment and whether there are any ecological impacts from this chronic release?

Upon entering the river, estuary or coastal water, sewage eﬄuent will be diluted. The resulting concentration of pollutants (antibiotics, metals, biocides, and ARGs) will interact with the native flora and fauna and begin to change the microbial community structure and genetic makeup (e.g., prevalence of resistance genes). Changes in the microbial community by means of any number of mechanisms, of which pollution (e.g., metals, biocides, and antibiotics) is but one, have been shown to have significant impacts on the aboveground diversity and functioning of terrestrial ecosystems (Wardle et al., 2004; Kong et al., 2006; Allison and Martiny, 2008; Bardgett and van der Putten, 2014).

The introduction of these pollutants into recreational and coastal bathing water, primarily through combined sewer overflows (CSOs), will elevate exposures to humans (Leonard et al., 2015) and by extension all wildlife that inhabits and feeds off/within the impacted water systems (e.g., shellfish or birds). However, little is known about the chronic effects from chemical exposure or the elevated prevalence of ARGs within a food web.

### Land Spreading of Manure and Biosolids

Human and animal use antibiotics, biocides, metals, and ARGs can all be found within products, such as: sewage sludge, anaerobic digestate, manure or slurry, which are spread upon agricultural soil for the purpose of fertilizing crops and/or improving soil properties. The dissemination of these materials on soil increases the ARG exposure risk to: (1) (wild) animals; (2) crops; (3) adjacent surface water bodies; (4) groundwater; (5) farm workers; and (6) air as dust particles from land spreading or aeolian erosion (Heuer et al., 2011; Ferro et al., 2015; Kyselková et al., 2015). The persistence and changes in the ‘resistome’ [the collection of genes that are capable of conferring resistance toward antibiotics when expressed in a susceptible organism (Dantas and Sommer, 2012)] of sludge or manure after it is anaerobically digested or composted is only recently emerging. A recent study demonstrated 156 unique ARGs and MGEs encoding resistance after composting sewage sludge, suggesting that the land spreading of composted sludge on a field will likely lead to the spread of ARGs in the soil and wider environment (Su et al., 2015). An increase in the prevalence of key resistance genes in the bacterial and bacteriophage fraction after digestion of sludge led the authors to similarly conclude that the agricultural use of treated sludge could contribute significantly to the spread of ARGs in the environment (Calero-Cáceres et al., 2014). The persistence of antibiotics in soil varies greatly in the literature between a few days (chloramphenicol, ceftiofur) to as high as 300 days (oxytetracycline, sarafloxacin) (Tasho and Cho, 2016). The persistence of antibiotics increases at low temperatures, unexposed to light (i.e., deeper soil layers), and often in high organic conditions, i.e., sludge or organic soils (Bondarczuk et al., 2016; Tasho and Cho, 2016). The fate of many antibiotics in soil will also be sensitive to pH and soil properties (Kümmerer, 2009).

The effects from land spreading of pig slurry on soil, as measured by increased prevalence of class 1 integrons (*intI1)*, has been shown to be maintained for several months after application (Byrne-Bailey et al., 2011). It has been suggested that the widespread use of land spreading of manure significantly contributes to the loading of some stretches of river and catchments, particularly in the U.S. where >100 million tons of feces and urine was attributed to a 65 million-head of hog per year industry (Sarmah et al., 2006; USDA, 2015a). The challenge of containing and disposing of feces is further impacted by substantial contributions from the cattle (95 million head of cattle in 2010) (USDA, 2010) and the poultry industries (8.5 trillion head of broiler chickens, 240 billion head of turkey) (USDA, 2015b).

The dissemination of metals from within sludge/manure on soil has been shown to lead to measurable changes in microbial community structure and in some cases declines in ecosystem services, such as nitrification (Chaudri et al., 1993; Halling-Sørensen, 2001; Walter et al., 2006; Fortuna et al., 2012). However, there is no evidence that antibiotics are phytotoxic at concentrations found in sludge amended to soil (Jjemba, 2002). Determining whether antibiotics and ARGs have an impact on soil ecosystem health is a key area for environmental regulators, which does not fall within the scope of human health impacts.

### Air Transmission (Bioaerosols)

Widespread manure distribution, either through feedlots or land spreading can facilitate the dissemination of ARGs (Aust et al., 2008; McEachran et al., 2015; Wang et al., 2015). In addition, several antibiotics have been recorded downwind of feedlots at concentrations similar to that found in rivers downstream of sewage outlets (0.5 to 4.6 μg/g). More broadly, antimicrobials, such as antifungals (e.g., azole fungicides), have significant aerosol dissemination pathways. Broadcast application of azole fungicides has likely facilitated the emergence of azole-fungicide resistance in *Aspergillus fumigatus* (a life threatening infection of the lungs, and a common component of composting bioaerosols) (Verweij et al., 2009). Strong evidence in support of an environmental origin of resistance can be found in the literature, where dominance of a single resistance mechanism in >69% of Dutch azole-resistant *A. fumigatus* isolates was recovered from epidemiologically unrelated patients, indicating an environmental driver of resistance such as the use of azole fungicides for plant and material protection (Snelders et al., 2008).

### Food Plants

A significant number of papers exist in the literature demonstrating the sensitivity and uptake of antibiotics from irrigation water, manure- or sludge-amended soils by crops into the plant biomass (Kang et al., 2013; Azanu et al., 2016). For example, onions, cabbage, and corn have been shown to take up chlortetracycline (Dolliver et al., 2007); corn, lettuce, and potato have been shown to take up sulfamethazine (Dolliver et al., 2007); wheat has been shown to take up chlortetracycline into the grain (Tasho and Cho, 2016); lettuces and carrot were shown to take up trimethoprim (Boxall et al., 2006). The degree to which this occurs in manure/sludge amended farms and the risk it poses to the environment remain poorly studied. The risk of phytoextracting antibiotics into a food products will be constrained by the mobility, bioavailability and persistence of antibiotics, which will be a function of the soil and its biological and physical properties. Only a small subset of all antibiotics in sludge and manure have been demonstrated to be phytoextracted into the plant biomass (e.g., sulfathiazole, sulfadiazine, sulfamethazine, sulfadimethoxine, chlortetracycline, and trimethoprim) (Tasho and Cho, 2016). The risk and implications to herbivores (e.g., insect, livestock, and human) from exposure to elevated concentrations of antibiotic in plants has not been well documented.

Of those antibiotics that are able to be phytoextracted, some have been shown to phytoconcentrate (Du et al., 2016). Those that phytoconcentrate can theoretically select for resistance in the microbiome of the plant as well as the organism that consumes/feed off the plant, despite apparently non-selective concentrations of antibiotics in the environment (Na et al., 2013; Azanu et al., 2016). A similar phenomenon has been shown for the phytoconcentration of metals, which cannot only theoretically impact the selection for metal resistance genes in the plant microbiome, but also in the herbivores that consume the plant (Winder et al., 1999). The phenomenon of bioaccumulation would need to be more explicitly understood if EQS’s were to be developed for antibiotics, as concentrations within organisms might greatly exceed MSCs or effect concentrations, even if environmental concentration were maintained below an EQS.

The effect of chronic antibiotic exposure on aquatic plants has been poorly characterized, but there is evidence to suggest that antibiotic exposed aquatic plants demonstrate significant changes in the phyllosphere microflora –a change that has unknown ecological consequences (Rico et al., 2014).

### Aquaculture and Shellfish Beds

Where there is veterinary use of antibiotics, biocides and/or metals in fish farms there will be increases in their environmental release, and concomitant selection for resistance genes. The accumulation and chronic exposure of river, estuarine and coastal environments to these AMR drivers can persist (Samuelsen et al., 1992) and spread AMR into and from the sediment (Sandaa et al., 1992). The World Organization for Animal Health (OIE) has developed standards in the Aquatic Animal Health Code on the responsible and prudent use of antimicrobial agents in aquatic animals as well as a List of Antimicrobials of Veterinary Importance (OIE – World Organization for Animal Health, 2015), which aims to optimize the balance between animal health needs and public health considerations. However, to date, there is no harmonized system of surveillance on the worldwide use and circulation of antimicrobial agents in aquaculture.

Antimicrobials are used in aquaculture to prevent and treat bacterial infections in fish and invertebrates (Cabello et al., 2013), however, in middle to low income countries, it can often be used to overcome what is considered sanitary shortcomings in fish rearing (Cabello, 2006). The UK reports the sale of 2 tons of antibiotics for use in the production of fish in 2014 (Veterinary Medicines Directorate, 2014). The 26 EU/EEA countries reported <10% of the total weight of food-producing livestock was from fish, with the exclusion of Norway, for which it represented 70% (European Medicines Agency, 2015). Canada, Norway and the U.S. permit aquaculture use of oxytetracycline, Canada and Norway permit use of florfenicol, and Norway permits aquaculture use of quinolones (Cabello et al., 2013). The quantity of antibiotic used per country can vary by as much as 175-fold for 1 ton of salmon (e.g., 0.008–1.4 kg of antimicrobial) (Cabello et al., 2013). The most abundant antibiotic (by mass) used in Norway for salmon production (821,997 MT in 2007), was a quinolone: oxolinic acid (681 kg in 2008), followed by florfenicol, a synthetic derivative of chloramphenicol (166 kg), and oxytetracycline (23 kg) (Burridge et al., 2010). Scotland’s salmon production (135,528 MT in 2007), by comparison, only used 9 kg florfenicol, and 75.4 kg of oxytetracycline in 2008 (Burridge et al., 2010). Norway, the largest producer of farmed Atlantic salmon in the world, had up until the implementation of new regulations in 2007 used much higher antibiotics: 1119 kg oxolinic acid, 5282 kg oxytetracycline and 302 kg florfenicol, in 2006 (Burridge et al., 2010).

The use and misuse of antibiotics in aquaculture has led to an increase in antibiotic resistance in fish pathogens, in the transfer of these resistance determinants to and from the sediment microbial community (Cabello, 2006; Cabello et al., 2013). Aquaculture has been shown to select for AMR in the fish microbiome and the surrounding environment (DePaola et al., 1988; Guardabassi et al., 2000; Schmidt et al., 2000, 2001). Changes in the fish microbiome have unknown consequences, presenting a significant knowledge gap (Guardabassi et al., 2000). The implications for the spread of AMR throughout the food web from fish-eating organisms has not been explored and is highlighted in the Knowledge Gap Section.

### Groundwater Quality

Antibiotics found in manure- or sludge-amended agricultural soils will enter groundwater as a result of rainfall, irrigation, and other human activities (Hirsch et al., 1999; Hu et al., 2010; Sui et al., 2015). A pan-European study of groundwater reported sulfonamide antibiotics in 24% of samples, reaching a maximum concentration of 38 ng/L, but averaging 2 ng/L (Loos et al., 2010). Among the higher concentrations of sulfonamides recorded in groundwater was from Spain, 3.4 μg/L (García-Galán et al., 2010). The biocides, i.e., triclosan, has been measured in UK aquifers in excess of 2 μg/L over a number of years (Stuart et al., 2012; Manamsa et al., 2016). Groundwater infiltration of veterinary pharmaceuticals from livestock wastewater impoundments has also been demonstrated, highlighting the role of livestock wastewater lagoons in groundwater pollution (Bartelt-Hunt et al., 2011). The implications for reduced groundwater depuration have been reported, where sulfamethazine and chlortetracycline was shown to inhibit the growth and denitrification of the nitrate present, thereby increasing the risk of nitrate pollution (Ahmad et al., 2014). Very little has been reported regarding the impact of antibiotic residues in groundwater on the generation of AMR in pathogens (Haznedaroglu et al., 2012).

### Ecological Health

The toxicological response of organisms to exposure to antibiotic biocides and metals goes beyond the scope of this review. However, there are intriguing reports in the literature that document ecologically relevant effects on a range of target organisms from exposure to sub-lethal concentrations of pollutants, including antibiotics. Measurable changes in the metabolites found in feces of mice have been reported after exposure to sub-lethal concentrations of antibiotics. In a study on mice, the metabolites were found to be critical for host physiology (e.g., steroid hormone synthesis) and an inflammatory response in the gut (Looft and Allen, 2012). Chronic exposure of some organisms to metals have been shown to either increase or decrease an immune response and cause autoimmunity problems (Fournié et al., 2001). The extent to which pollution-induced autoimmunity occurs in wildlife is unclear, however, where it does occur it is likely to have a measurable effect on the life-cycle and ecosystem services of that organism (Sattar et al., 2007).

In the case of plants, there is evidence to suggest that seed germination, root elongation and overall plant health can be sensitive to sub-inhibitory concentrations of antibiotics and metals in the soil (Pan and Chu, 2016). Exposure to sub-lethal concentrations of pollutants such as antibiotics, biocides, and metals can induce pollution-induced parasitization, which in turn has been shown to increase susceptibility to the toxic effects of the pollutant (Khan and Thulin, 1991). It is unclear how reproducible this linkage is between exposure and parasitization, but it has been suggested that parasites of aquatic organisms might potentially be used as indicators of pollution (Khan and Thulin, 1991). This same relationship between pollution exposure (particularly metals) and increased susceptibility to infection has also been shown to occur in plants (Sattar et al., 2007). There is the suggestion that pollution might have contributed to the observed increase in infections and abnormalities in amphibians, perhaps facilitated by host immune system effects or enhanced by persistent chemical pollutants acting in synergy or directly on the microbial pathogens to increase their persistence and virulence (Carey, 2000; Kiesecker, 2002; Sattar et al., 2007).

Bactericidal antibiotics, including β-lactams, quinolones, and aminoglycosides, can stimulate bacteria to produce reactive oxygen species (ROS), which are highly deleterious molecules that can interfere with the normal functions of oxygen-respiring organisms (Kohanski et al., 2010). ROS, such as hydroxyl radicals, are mutagens, which activates the error-prone SOS response and error-correcting repair systems. The induction of this DNA damage and repair cascade by low levels of antibiotics leads to an increase in mutation rates, which can result in the emergence of multidrug resistance (Kohanski et al., 2010). Hence, the mere presence of these radial oxygen species-inducing chemicals in the environment ensures that spontaneous mutations will occur at a greater rate and that antibiotic resistance can form *de novo* (Isidori et al., 2005; Kohanski et al., 2010; Dwyer et al., 2014).

## What are Our Key Knowledge Gaps?

It has been noted that the wider problem of AMR has many parallels to that of climate change, as they are both, arguably, an interdisciplinary, complex, global “tragedy of the commons” (Foster and Grundmann, 2006; Woolhouse and Farrar, 2014; Woolhouse et al., 2015). Many hundreds of researcher years have gone into producing our current understanding of the mechanisms of AMR selection and transmission (Aminov, 2010). However, despite this deep knowledge base, we are still left unable to answer many rather fundamental questions about the ecology and drivers of AMR in the environment (Huijbers et al., 2015). Given the urgency of mitigating AMR, there is a need to prioritize our knowledge gaps into two categories: those that will inform policy and those that will not. A pragmatic view of research priorities is not novel, as funding bodies have ‘Directed Research Calls’ that often specify ‘what we need to know.’ However, the evidence suggests that these calls have been both limited in number (relative to the importance of the topic) and devoid of questions that pertain to environmental health. A recent report that conducted a systematic observational analysis of antibacterial resistance research funding, showed that only 3% of research projects on antibiotic resistance proposed to tackle issues that relate to the environment (Kelly et al., 2016). One possible explanation for the dearth of research is that real world (field scale) environmental microbiology is messy and difficult to reproduce, and therefore seen less sympathetically by traditional grant review panels that tend to favor highly replicated and controlled studies –something rivers and WWTPs are not. A mechanism to prioritize the global research agenda on AMR and the environment does not yet exist; however, leadership from existing global bodies such as the WHO (World Health Organization), FAO (Food and Agriculture Organization) and OEI (World Organization for Animal Health) will be key, to ensure integration with existing global (World Health Organisation, 2015) and national (Department of Health/Defra, 2013) AMR Action Plans.

The following is a first attempt to shortlist the knowledge gaps that emerge from a review, with a focus on those which could directly inform policy.

(1) **Are there direct or indirect implications to the health, reproduction or ecosystem services of organisms or populations resulting from chronic exposure to elevated AMR drivers, as in **Figure 1**, in the environment?** Sub-question: (a) Can exposure to elevated AMR drivers increase susceptibility to infection, and how does this differ with age, gender, habitat quality, exposure time, mixture exposures?; (b) Are there implications to the environment from chronic exposure to elevated levels of ARGs and the MGEs in which they might be contained? (c) Do antibiotics, biocides or metals bioaccumulate and what are the implications for AMR selection, maintenance, and benchmarking regulatory thresholds?
(2) **What are the relative contributions of the different AMR pathways, as in **Figure 1**, for establishing, maintaining and disseminating ARGs in the environment?** Sub-question: (a) Are there temporal, spatial, or physical/geological factors that impact on the selection, maintenance, and spread of AMR in the environment? (b) Should pathways be prioritized for mitigation based solely on potential impact, or should it include tractability (i.e., “easy win” vs. greater impact)?
(3) **What are the relative contributions of the different AMR drivers, as in **Figure 1**, for establishing, maintaining and disseminating ARGs in the environment?** Sub-question: (a) How does ARG co-selection operate with mixtures of antibiotics, biocides and metals (e.g., concentration addition, independent action, synergism or antagonism)?; (b) Can understanding co-selection help define targets for mitigation–is this a tractable problem?; (c) Do bacteriophage play a meaningful role in the maintenance and transmission of ARGs in the environment? (Subirats et al., 2016); (d) What are the sources of ARG-containing phage–is mitigation of phage tractable?
(4) **What concentrations of AMR drivers are relevant for assessing the risk of AMR selection and co-selection?** Sub-question: (a) What is a useful benchmark for assessing risk and the success of any mitigation/intervention measures (e.g., MIC, MSC, ARG prevalence, ecological cutoff/break points?); (b) What is the most appropriate time period for assessing the success of mitigating measures?
(5) **Are there direct or indirect implications from the trophic transfer of antibiotics, biocides, metals, or ARGs found within microorganisms, animals (aquaculture), or plants?** Sub-question: (a) Are some chemicals more susceptible to uptake (i.e., ionisable chemicals) in plants and do these pose the greatest risk to organisms and ecosystems?; (b) What are the effects of pH on the ionization of antibiotics and their differential toxicity to different target organisms?; (c) What role does metal speciation and metal bioavailability play in AMR selection and maintenance?; (d) How does antibiotic bioavailability in soil influence exposure and risk?

These knowledge gaps have been, in part, summarized in Eq. 1:

(1) $$\begin{array}{c} E=\sum_{i-1}^{n}\sum_{j-1}^{m}cij \end{array}$$

where c_ij_ = the *j*-th of n chemicals with similar modes of action from the *i*-th of m pathways. *E* is the therefore the sum total of all chemicals with similar modes of action from different pathways per site. It is the aspiration to use minimum selection concentrations (MSC) derived from complex microbial communities in different environments (e.g., aquatic, sediment, soil, sewage, crops, and wildlife) to calculate a risk coefficient (R):

(2) $$\begin{array}{c} R=\frac{E}{MSC} \end{array}$$

where *R* > 1 represents a high risk of ARG selection and *R* < 1 represents a lower risk. An evidence-based determination (estimation) of the ARG-selection risk at each location can better inform and prioritize subsequent mitigation efforts.

## Thought Experiment

It has been widely shown that ARGs are a natural phenomenon, and that we have been living with ARGs in the environment (and within our microbiome), since the dawn of our species (D’Costa et al., 2011). It has been shown quite clearly in the literature, that there has been a substantial increase in the prevalence and diversity of ARGs since the time of mass production of antibiotics (Knapp et al., 2010; Graham et al., 2016). So, is the problem with AMR in the environment simply a matter of increasing prevalence of ARGs? If it is a matter of prevalence, does prevalence of ARGs only have meaningful implications to humans and farmed animals? As previously discussed, there are numerous studies that show direct toxic or metabolic effects from exposure to the drivers of ARGs–this is not really debated. Whether these effects are meaningful to the population, community or ecosystem and its services is where our knowledge gap lies. Most studies focus on the organism; based on that sub-set of life on our planet, the answer is yes, there are measurable meaningful effects on many organisms to environmentally relevant concentrations of antibiotics, biocides and metals. However, it has been argued that some meaningful effects found at an organismal level might not translate into meaningful implications to the population, community, ecosystem and its services (Sumpter, 2009; Johnson and Sumpter, 2016). There is a lack of research asking this “meaningful” level of impact at the appropriate scale–the ecosystem. The following four Thought Experiments, follow on with these critical questions, with the aim to highlight the obligate need for a critical interpretation of ‘impact’ and a pragmatic and holistic vision for mitigation where these are tractable, meaningful and efficacious.

### Thought Experiment I

The decision by the European Commission to include macrolides on the Watch List indicates that their environmental impact, based on classical toxicity studies, might be sufficiently high to justify their regulation. Will regulating macrolides contribute to a reduction in AMR? Where macrolides compose the majority of AMR drivers in a habitat, one might expect it could be impactful in reducing the prevalence of ARGs. However, there are few scenarios where this condition is met–possibly downstream a macrolide manufacturing facility. The reality is that most antibiotics enter the environment through sewage eﬄuent or from manure/biosolid-amended soil, all of which are rich in a wide range of AMR drivers (e.g., antibiotics, biocides and metals). Hence, it is unrealistic to expect macrolide regulation to substantially lower ARG selection. Establishing EQS’s for macrolides should not be interpreted as a mitigation measure for AMR since there is no evidence to date to suggest these two goals are necessarily linked. Moreover, regulating macrolides will not eliminate their use, but simply lower their use and subsequent concentration the environment. The question remains whether a reduction in macrolide release into the environment to satisfy an EQS will reduce macrolide-ARG prevalence.

### Thought Experiment II

If the selection of ARGs within WWTPs is an important contributor of ARGs to the environment, then it might be of limited consequence that a WWTP employs technologies such as: activated carbon adsorption, advanced oxidation processes, nanofiltration, reverse osmosis, and membrane bioreactors (Luo et al., 2014), to target the removal of macrolides from its eﬄuent, as the ARGs have already been enriched within the treatment process and are being chronically released into the environment. There are no wastewater treatment technologies in widespread use that remove ARGs to zero. This scenario highlights an important knowledge gap: What is the relative importance of antibiotics, biocides, metals and ARGs for maintaining and disseminating ARGs in sewage works and the environment (Knowledge Gap 3)? If the release of ARGs into the river are deemed relatively unimportant, then there is more reason to suspect that the removal of metals, biocides or antibiotics could be useful. However, if metals are important relative to the other drivers, then treatment technologies will need to address this challenge. Technologies used to remove metals (Fu and Wang, 2011), such as chemical precipitation, ion-exchange, adsorption, membrane filtration, coagulation–flocculation, flotation and electrochemical are unlikely to be effective in reducing antibiotics, biocides or ARGs. Hence, defining the relative importance of resistance-driving chemicals to selection and dissemination of ARGs is key–as each will necessitate a different solution at the WWTP. In the case of ARGs, it will be important to discern the relative role of bacteriophage in the dissemination of ARGs, as they represent a nano-size particle that protects the ARGs held within it, making it very difficult to remove from the waste stream.

### Thought Experiment III

Emerging from Thought Experiments I and II is the idea that WWTPs are a major pathway for dissemination of AMR drivers. WWTPs are a convenient location for mitigating AMR and its drivers, as it represents the aggregation of many sources of resistance-driving chemicals and ARGs. However, addressing the AMR challenge at the point of the WWTPs comes with a very significant cost. It has been estimated that the UK water industry would require €30 billion in investment to comply with proposed EC limits on ethinyl estradiol (EE2, ‘the pill’) (Owen and Jobling, 2012). Regulations on the release of macrolides in eﬄuent would likely garner a similar price tag. If solutions needed to be developed for substantially reducing levels of antibiotics, biocides, metals, and ARGs, it would greatly increase the cost of treating wastewater. Improvements in wastewater treatment to tackle the drivers of AMR can also offer reductions in a wide diversity of contaminants of emerging concern, including pathogens and viruses (Huijbers et al., 2015). As such, improvements to WWTPs might be more easily justified to the public in light of the broader benefits to society relative to the costs. In addition to upgrading WWTPs, there is reason to consider ‘upstream’ (i.e., close to source) mitigation measures (see Thought Experiment IV). It is also important to note that the UK water and sewerage industry is highly regulated to ensure the cost of bills is affordable. To that end, Ofwat (The Water Services Regulation Authority, the body responsible for economic regulation of the privatized water and sewerage industry in England and Wales) negotiates the research and investment budget with the water companies many years in advance. Work to develop the 2020–2025 business plans of all water and sewerage companies begins in 2016. Hence, there is a need to identify cheaper and quicker ‘wins,’ as the regulatory constraints on research and investment, the reluctance to exceed a maximum acceptable cost to the taxpayer, and the speed at which the industry is capable of responding are hugely limiting of any quick fixes.

### Thought Experiment IV

If the solution to mitigating AMR in the environment must move ‘upstream’ from the WWTP, it likely becomes the remit of several additional government regulators. AMR Action Plans might benefit from adapting the philosophy of the recycling movement “reduce, reuse, recycle” as it offers a more holistic and cost-effective mechanism for a reduction in the load of resistance-driving chemicals entering the waste stream. Without knowing the relative role of each of the drivers of AMR (antibiotics, biocides, metals, and ARGs), it would be necessary to identify, quantify and reduce each of the chemical drivers entering the wastewater. There are many activities currently underway on a global scale that aim to tackle some of these upstream sources of AMR drivers, e.g., reducing antibiotic misuse in human and veterinary use; reducing biocide use in personal care and household products; capture, reuse and recycle metals within the waste stream. The monumental task that this represents highlights the need to prioritize research to address these Knowledge Gaps.

## Concluding Thoughts

The relevance of the AMR pathways and drivers to the environmental regulator can be crudely summarized in three, high level, points:

(1) Low levels of antibiotics, metals, and biocides can directly select (and co-select) for ARGs within the AMR pathways that are under the remit of the Environment Agency, i.e., sewage eﬄuent, rivers, sludge, irrigation system, (bio)aerosols, aquaculture, industry, and groundwater.
(2) Pathways for the dissemination of antibiotics, metals, biocides, and ARGs into animals, plants, food (animal, plant and shellfish), groundwater, rivers and bathing waters, are within the remit of the Environment Agency.
(3) Sub-lethal effects of antibiotics, metals and biocides on organisms, their microbiome and ecosystem services (e.g., rivers, coastal waters, and soil) can impact the health, yield and safety of economically important food products and the wider biome.

It is clear that environmental regulators should have an important role to play in future efforts to mitigate the environmental and human health risks from AMR. However, it is also apparent, that they represent only one of many stakeholders that can contribute to the solution(s). Implementation of ‘upstream’ solutions, such as ‘reduce, reuse, recycle,’ will necessitate coordinated strategies across multiple regulatory bodies and stakeholders, including: *Department of Health* [(including Medicines and Healthcare products Regulatory Agency, NHS England and Public Health England), Food Standards Agency], *Department of Food, Agriculture and Rural Affairs* (Defra) [including: Centre for Environment, Fisheries and Aquaculture Science, Water Services Regulation Authority (Ofwat)], Veterinary Medicines Directorate, Environment Agency (equivalent regulator in adjacent countries: Scottish Environmental Protection Agency, Natural Resources Wales, Northern Ireland Environment Agency) as well as, Water and Sewerage Companies, industry and wider society. The first step toward this vision for a holistic AMR mitigation strategy, is for each of the stakeholders to critically evaluate the role they play in contributing to the composition and dissemination of resistance-driving chemicals. This document reflects a first effort to that end in England and the wider U.K.

It is important to recognize that the lack of environmental focus in AMR Action Plans and the O’Neill Review is, arguably, because: (1) our understanding of AMR in the environment is so unsatisfactory that there is very little that can be suggested for mitigation without employing the precautionary principle as the primary rationale for action, and (2) the disciplines of pollution, environmental microbiology, and the wider infectious disease fields have been chronically underfunded (Head et al., 2013; Bragginton and Piddock, 2014; Kelly et al., 2016; Piddock, 2016), leaving them unable to provide the evidence base needed to inform policy. Unless society is prepared to assume the non-trivial costs associated with a precautionary approach, e.g., increased water bills to pay for substantial upgrades in WWTPs, there will be a need to prioritize the research that addresses the ‘Knowledge Gaps,’ as previously detailed. The point should not be lost that such an investment in upgrading WWTPs might allow for multiple ‘victories,’ through the co-removal or reduction of other pollutants and hazards.

However, there is reason to be optimistic, as for the first time, all seven UK Research Councils have come together to collaborate on funding long-term cross-disciplinary, integrated research on AMR (Medical Research Council, 2014). An Antimicrobial Resistance Funders’ Forum has also been established to provide a framework for delivering a more coordinated research portfolio and to maximize the impact on both a national and international policies and activities.

## Author Contributions

AH, VR, and AS conceived of the review outline. AS researched and drafted the manuscript. HS provided significant input to several sections to improve clarity and accuracy.

## Conflict of Interest Statement

The authors declare that the research was conducted in the absence of any commercial or financial relationships that could be construed as a potential conflict of interest.
